# Epigenetic regulation of *SYNGAP1* in alcohol use disorder in whole blood and saliva

**DOI:** 10.3389/fpsyt.2025.1661760

**Published:** 2025-11-06

**Authors:** Susanne Edelmann, Christine Kummer, Sarah Pasche, Milan Zimmermann, Vanessa Nieratschker

**Affiliations:** 1 Department of Psychiatry and Psychotherapy, University Hospital of Tuebingen, Eberhard Karls University of Tuebingen, Tuebingen, Germany; 2 German Center for Mental Health (DZPG), Tuebingen, Germany

**Keywords:** alcohol use disorder (AUD), SYNGAP1 gene, DNA methylation, epigenetics, validation

## Abstract

Epigenetic regulation is significantly altered in individuals with alcohol use disorder (AUD), representing a promising avenue for understanding its pathomechanisms and developing new therapies. In an earlier epigenome-wide study of CD3+ T cells, we identified *SYNGAP1*–a critical regulator of synaptic plasticity that influences neuronal communication and network remodeling–as epigenetically dysregulated, with significantly lower DNA methylation (DNAm) in patients than controls. After three weeks of inpatient withdrawal, *SYNGAP1* DNAm increased to control levels. In the present study, we aimed to validate these differential *SYNGAP1* DNAm levels in an independent cohort of 64 AUD patients and 83 healthy controls in peripheral blood and saliva, to assess its potential as a biomarker. Using a linear mixed-effects model including AUD status and covariates, no significant differences were observed. *Post hoc* analyses revealed an unexpected pattern: In blood, *SYNGAP1* DNAm was higher in patients before treatment than controls, with no difference after withdrawal; in saliva, no differences or therapy effects were detected. Overall, these results did not confirm our previous findings, suggesting limited value of *SYNGAP1* DNAm as a biomarker for AUD. While blood methylation showed some association, the effect direction contradicted earlier results, and saliva showed no signal. Further research is needed to clarify *SYNGAP1* epigenetic regulation in AUD and its potential relevance for biomarkers or therapy.

## 1 Introduction

Alcohol Use Disorder (AUD) is a severe chronic disorder contributing substantially to the global burden of disease (1). The development of AUD underlies both genetic and environmental factors (2, 3), and gene-environment interactions, such as epigenetic mechanisms, play a pivotal role (4). Epigenetics describes the – reversible – modulation of genomic activity and gene function without changing the DNA sequence itself. One of the most studied epigenetic mechanisms is DNA methylation (DNAm) (5). AUD has been widely described as being associated with altered DNAm (4, 6, 7). Several investigations conducting epigenome-wide association studies (EWAS) in blood and brain provided evidence for altered DNAm patterns, e.g. in genes involved in glutamate signaling (8), immune-related pathways (9, 10), and glucocorticoid and inflammation-related signaling (11). Recently, White et al. (2024) identified 105 AUD-associated CpGs annotated to 120 genes within and across brain regions that were enriched in histone marks tagging active promoters (12). In a previous epigenome-wide study in our group, we identified decreased DNAm levels of the CpG site cg02652579 present in the promotor region of *Synaptic Ras-GTPase-activating protein* gene (*SYNGAP1*) in CD3^+^ T-cells of male AUD patients compared to matched control individuals. Interestingly, following three weeks of inpatient withdrawal treatment, *SYNGAP1* DNAm levels increased and reached levels observed in healthy control individuals (13). *SYNGAP1* encodes for the SynGAP protein (14, 15) which is part of complex networks located on the postsynaptic density (PSD), mainly in the cortex and hippocampus. SynGAP fulfills several functions in neurotransmitter signaling, morphology of synapses and scaffolding of protein networks (15, 16). Furthermore, SynGAP promotes, via various intracellular signal cascades, AMPAR (*α-amino-3-hydroxy-5-methyl-4-isoxazolepropionic acid receptor)* insertion and long-term potentiation (LTP) induction in activated neurons while providing for a stable number of AMPARs during baseline activity (15, 17–23). An association between alcohol consumption and *SYNGAP1* has been described recently in mice, where SynGAP protein was significantly downregulated in animals undergoing alcohol withdrawal (24).

The aim of the current study was to validate our earlier finding of significantly altered DNAm patterns of *SYNGAP1* (i.e. cg02652579) in more easily accessible somatic tissue – peripheral venous whole blood and saliva – as well as female AUD patients. *SYNGAP1* was prioritized for validation as it was among the top hits exhibiting this therapy-associated reversal pattern, suggesting its potential involvement in AUD pathophysiology and response to treatment. Therefore, we investigated the potential of *SYNGAP1* DNAm to serve as a novel epigenetic biomarker for AUD diagnosis as well as withdrawal therapy outcome. Our study may support the understanding of underlying molecular processes, which could open new perspectives on SynGAP as a possible therapeutic target, enabling personalized therapy options and a more effective health care.

## 2 Methods

### 2.1 Study subjects

In total, 147 participants were included in the study between 2020 and 2023. The patient group consisted of 64 individuals diagnosed with a severe form of AUD (Alcohol dependence) according to the International Statistical Classification of Diseases and Related Health Problems, 10th Revision (ICD-10 (25),). Patients underwent a three-weeks inpatient qualified withdrawal treatment according to the German S3 guideline on alcohol related disorders (26) at the Department of Psychiatry and Psychotherapy of the University Hospital Tübingen. Samples and data have been collected at hospital admission (T1), as well as after three-weeks of therapy (T2). Samples and data of 83 control individuals have also been collected. At T2, 134 participants (N = 53 AUD patients, N = 81 healthy control individuals, **Supplementary Table S1**) remained in the study. Of both groups, individuals with comorbid substance use disorder other than nicotine or alcohol and with comorbid psychiatric disorders other than Major Depressive Disorder were excluded. At both time points, the following self-administered questionnaires were assessed: alcohol consumption using the Alcohol Use Disorder Identification Test (AUDIT (27),) for alcohol consumption and Obsessive-Compulsive Drinking Scale (OCDS (28),) for alcohol craving (**Supplementary Table S1**).

All participants were of European descent and aged between 20 and 71, sampling numbers and details are shown in **Supplementary Table S1**. They provided informed written consent. The study was approved by the ethics committee of the University of Tübingen (Reference number 264/2018 BO2) and was conducted in accordance with the Declaration of Helsinki.

### 2.2 DNA methylation analysis

Ethylenediaminetetraacetic (EDTA) blood and saliva samples (in Oragene^®^ DNA Collection Kits, DNA Genotek, Ottawa, Ontario, Canada) were collected at both time points (T1 and T2). The DNA was extracted from blood samples using the QIAamp^®^ DNA Blood-Maxi Kit (Qiagen, Hilden, Germany) and with Oragene^®^ prepIT•L2P (DNA Genotek, Ottawa, Ontario, Canada) for saliva samples, respectively, according to the manufacturer’s instructions. The DNA was stored at -20 °C until proceeding and bisulfite converted with EpiTect^®^ Fast DNA Bisulfit Kit (Qiagen, Hilden, Germany). The region of interest within the promotor region of *SYNGAP1* (hg19, chr6:33386818-33387117) was amplified using the PyroMark PCR Kit (Qiagen, Hilden, Germany) according to the manufacturer’s instructions. PCR primers (Metabion, Planegg, Germany) were as follows: PCR forward primer: 5 ´-GAG GGG TTA ATG AGA GGT AGA GAG GTG-3 ´; PCR reverse primer: Biotin-5’- - CCC CAC TTC CCT ACC CTA AAA CC -’3. The PCR products were quality-controlled on an agarose gel and subsequently pyrosequenced with the PyroMark^®^ Q24 using the Pyromark Gold Q24 reagents (Qiagen, Hilden, Germany) and the following sequencing primer: 5’-TGG TTT GGT GGT GGG GAT GTT-3’. The analyzed CpG site (cg02652579) is located at chr6:33386967 (hg 19). The DNAm level was analyzed using the PyroMark^®^ software (Version Q24 2.0.7). At least two replicates of the PCR and sequencing reaction were performed for each sample. Only replicates with a deviation of ≤ 3% between runs were further analyzed. In all steps of the protocol, samples were arranged in a balanced order to avoid batch effects.

### 2.3 Statistical analysis and visualization

All analyses were performed using the software environment R and Python. Statistical tests, that are available within the R package ggpubr (version 0.6.0) (29) or the Python package stat.test (30) were used depending on the analysis specified in the following sections.

Distribution of the values per group, variable (such as age and questionnaire scores) and time point of sampling was analyzed applying the Shapiro-Wilk-test (**Supplementary Table S2**). To investigate the effects of AUD and its therapy on *SYNGAP1* DNA methylation levels, a linear mixed-effects model (using the R package lme4 (31)) was fitted including age, sex and smoking as covariates using the following formula: DNAm ~ group*time + group*smoking + age + sex + (1|ID).

For the *post-hoc* tests, normally distributed values (i.e., DNAm data of blood samples) were analyzed with parametric student´s t-test. Non-parametric tests (Mann-Whitney U test for independent samples and Wilcoxon signed rank test for paired data) were applied for not-normally distributed data. Benjamini-Hochberg correction (32) was performed to correct for multiple testing and therefore, protect against false positive or Type 1 errors. An adjusted p-value was calculated for the respective number of tests for time-wise demographic/clinical variables as well as DNAm data of blood and saliva independently. An adjusted *p*-value (*p.adj.*) <.050 was considered as significant. Effect sizes were calculated using Cohen’s d (33).

## 3 Results

The study sample included 64 AUD patients and 83 healthy control individuals (**Table 1**, **Supplementary Table S1**). Although age, sex and smoking behavior of both groups were aimed to be matched throughout the recruitment process, the two groups still revealed significant differences: Healthy control individuals (HC) were significantly younger (40.64 ± 13.73, W = 3708, *p.adj.* < 0.001, **Table 1**) and included more females (66% females, X-squared = 9.71, df = 1, *p* = 0.002, **Table 1**) than patients (age: 49.8 ± 11.47 years, 39% females, **Table 1**). Although assessed, it was not possible to match the groups for smoking status resulting in a large overlap of the variables AUD status and smoking status (77% of the AUD patients were smokers and 95% of the healthy control group were non-smokers, X-squared = 74.38, df = 1, *p* < 0.001).

**Table 1:** Demographic and clinical information of the study cohort.

Variable		Group		*p.adj.*
		AUD patients (N = 64)	Healthy control individuals (N = 83)	
Sex (Females)		n = 25 *(39%)*	n = 55 (*66%*)	0.002
Smoking (Yes)		n = 46 (*77%*)	n = 4 (*5%*)	< 0.001
Age (years)		49.80 ± 11.47	40.64 ± 13.73	< 0.001
AUDIT	T1	26.40 ± 7.95	2.93 ± 2.30	< 0.001
	T2	22.90 ± 8.48	3.09 ± 2.39	< 0.001
OCDS	T1	21.30 ± 7.60	2.00 ± 2.66	< 0.001
	T2	13.30 ± 6.21	1.63 ± 2.24	< 0.001

AUDIT scores were significantly higher in patients at both time points (T1: AUD: 26.40 ± 7.95, HC: 2.93 ± 2.30, p < 0.001; T2: AUD: 22.90 ± 8.48, HC: 3.09 ± 2.39, W_T1_ = 4573, *p.adj.*_T1_ < 0.001; W_T2_ = 1064, *p.adj.*_T2_ < 0.001; **Table 1**). OCDS scores were also significantly higher at both time points (T1: AUD: 21.30 ± 7.60, HC: 2.00 ± 2.66, W_T1_ = 4946, *p.adj.*_T1_ < 0.001; T2: AUD: 13.30 ± 6.21, HC: 1.63 ± 2.24; W_T2_ = 4059, *p.adj.*_T2_ < 0.001; **Table 1**), which shows elevated craving and obsessive tendencies towards alcohol in AUD patients. All questionnaire scores significantly improved post therapy in patients (OCDS: V = 1154, *p.adj.* < 0.001 (n_T1_ = 60, n_T2_ = 52); AUDIT: V = 198, *p.adj.* = 0.027 (n_T1_ = 56, n_T2_ = 24)), showing a tendency of positive effects of the detoxification treatment on drinking behavior and withdrawal of AUD patients.

To investigate the effects of AUD and its therapy on *SYNGAP1* DNAm in blood while accounting for potential effects of demographic variables, a linear mixed-effects model with the factors group (AUD patients vs. healthy control individuals), time (pre and post withdrawal treatment) as well as smoking status and their interaction together with age and sex was fitted. A significant effect of time was revealed (Std. Error = 0.366, *p* = 0.012, **Supplementary Table S3**). However, neither a significant effect of AUD status (Std. Error = 1.202, *p* = 0.758, **Supplementary Table S3**) nor of the interaction of AUD status and time (reflecting withdrawal treatment, Std. Error = 1.108, *p* = 0.488) was observed.

As previously noted, unfortunately, smoking status was strongly overlapping with AUD status in the cohort (**Table 1**). To address this, we included both smoking status and the interaction between AUD and smoking status in the model. However, neither smoking status (Std. Error = 1.918, *p* = 0.163) nor the interaction term reached significance (Std. Error = 1.098, *p* = 0.644, **Supplementary Table S3**). Furthermore, neither age (Std. Error = 0.026, *p* = 0.611) nor sex (Std. Error = 0.736, *p* = 0.556) had a significant effect on *SYNGAP1* DNAm in blood.

**Figure 1:**
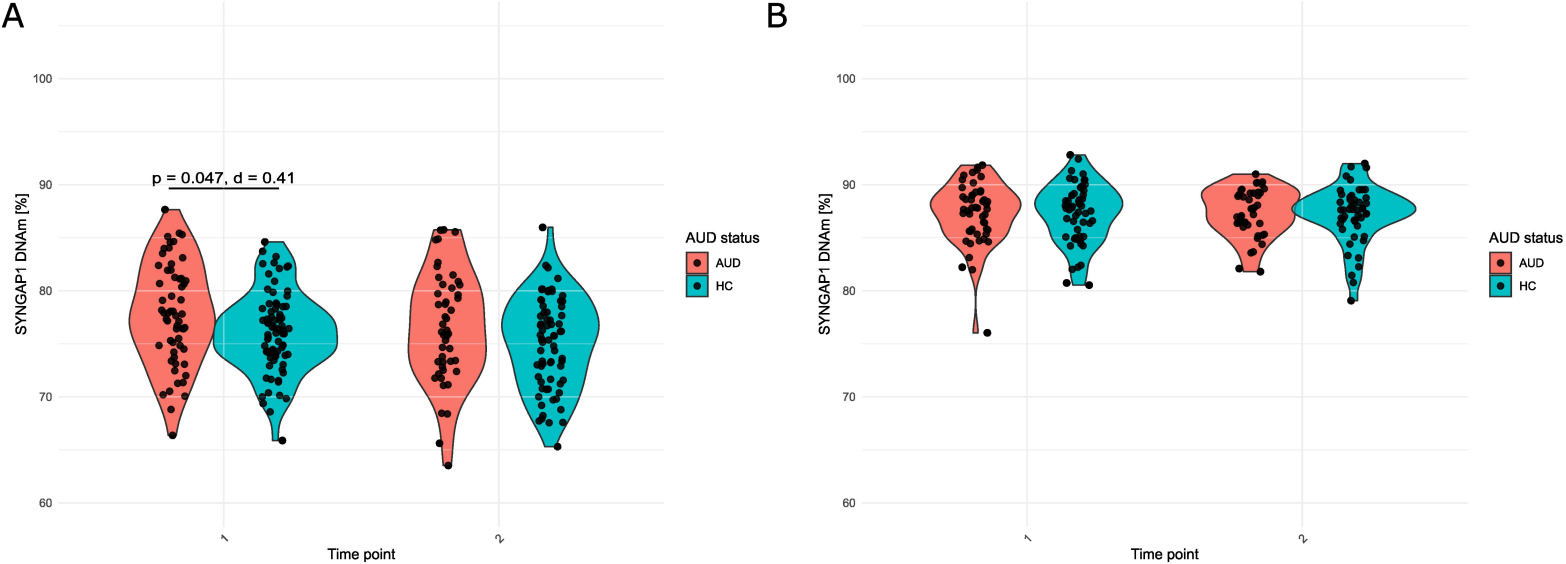
*SYNGAP1* DNAm (%) in **(A)** Blood and **(B)** Saliva of patients (AUD) and healthy control individuals (HC) at T1 and T2. For A, student’s t-test with Benjamini-Hochberg correction was used. For B, Mann-Whitney U test with Benjamini-Hochberg correction was used. For A, Cohen’s d is additionally reported.

However, *post-hoc* tests comparing AUD patients and healthy control individuals revealed that prior to the three-weeks inpatient withdrawal treatment, *SYNGAP1* DNAm of patients was significantly higher with an average of 77.8 ± 4.78% compared to that of healthy control individuals with an average of 76.1 ± 3.77% at T1 (t = 2.30, *p.adj.* = 0.047; Cohen’s d = 0.41, AUD patients: n = 64, Healthy controls: n =83, **Figure 1A**).

After the three-weeks inpatient withdrawal treatment (T2), *SYNGAP1* DNAm of AUD patients remained without significant changes compared to T1 (t = 1.20, *p.adj.* = 0.237; Cohen’s d = 0.16, n = 46). Also, in healthy control individuals, *SYNGAP1* DNAm levels in blood did not significantly change compared to T1 (t = 1.71, *p.adj.* = .184; Cohen’s d = 0.18, n = 72). However, the difference in DNAm between the groups at T1 was no longer observed at T2 (t = 1.68, *p.adj.* = 0.097, Cohen’s d = 0.32).

The same way, we analyzed *SYNGAP1* DNAm in saliva of AUD patients in comparison to healthy controls before and after withdrawal treatment. *SYNGAP1* DNAm of saliva was in average higher compared to blood *SYNGAP1* DNAm (DNAm_saliva(AUD)_: 87.3 ± 2.91% and DNAm_saliva(HC)_: 87.3 ± 2.78% at T1). The linear mixed effects modelling did not reveal any significant effects of AUD status or any other tested variable (smoking, age, sex and time as well as the interaction of AUD status and time or smoking status, respectively) on saliva DNAm (**Supplementary Table S3**). Replacing AUD status with smoking status in the model revealed similar results (**Supplementary Table S4**).

Posthoc tests furthermore confirmed stable saliva DNAm levels throughout treatment (DNAm_saliva(AUD)_: 87.4 ± 2.26% and DNAm_saliva(HC)_: 87.2 ± 2.72% at T2 compared to T1 mentioned before; W_AUD_ = 458, *p.adj.*_AUD_ = 0.700, Cohen’s d_AUD_ = 0.14, W_HC_ = 585, *p.adj.*_HC_ = 0.830, Cohen’s d_HC_ = 0.00) without an influence of AUD (W_T1_ = 1388, *p.adj.*_T1_ = 0.921, Cohen’s d_T1_ = 0.00, W_T2_ = 1064, *p.adj.*_T2_ = 0.921, Cohen’s d_T2_ = 0.07, **Figure 1B**).

## 4 Discussion

In the present study, we investigated DNA methylation of a CpG site (cg02652579) in the promotor region of *SYNGAP1* in whole blood and saliva of AUD patients in a longitudinal study design – before (T1) and after (T2) a three-week inpatient withdrawal treatment – compared to healthy control individuals. Analyzing *SYNGAP1* DNAm in whole blood of 64 AUD patients and 83 controls, we could not confirm our previous results of lower DNA methylation levels in CD3^+^ T cells in patients than in control individuals. While a linear mixed-effects model including AUD status and relevant covariates, revealed no significant differences in *SYNGAP1* DNAm, *post-hoc* analyses showed higher *SYNGAP1* DNAm in patients prior to treatment compared to controls. After withdrawal therapy, this difference was no longer evident. In saliva, no significant differences in *SYNGAP1* DNAm were detected between groups, and therapy showed no effect. Altered DNAm in association with AUD has been shown before by several studies on an epigenome-wide ( (8, 9, 34) as well as candidate gene level (35, 36). In a previous epigenome-wide study, we showed reduced methylation of the same CpG site (cg02652579) associated with *SYNGAP1* in CD3^+^ cells of AUD patients (13). Moreover, *SYNGAP1* expression has been identified to be correlated with alcohol withdrawal in mice brains (24). Interestingly, Witt et al. (2022) observed a significantly hypomethylated CpG site (cg07573985), which is 500 bp upstream of cg02652579, in blood of AUD patients (37). Although they measured hypomethylation rather than the hypermethylation we identified for CpG site cg02652579, their data support the notion that *SYNGAP1* DNAm is influenced by AUD.

Statistically significant effects of the three-week inpatient withdrawal therapy on the blood DNAm levels were not observed. Therefore, a potential dysregulation of *SYNGAP1* on the DNA methylation levels as revealed by the groupwise *post hoc* test could be either consistent or – as the differential methylation of *SYNGAP1* observed at T1 was no longer present at T2 – the small size of our sample does not allow definitive conclusions, but leaves the trend of reversing towards healthy levels after therapy. Brückmann et al. identified such a reversal of cg02652579 methylation in their epigenome-wide approach in CD3^+^ cells of AUD patients undergoing withdrawal therapy, although in this case, the initially lower methylation increased with therapy approximating the healthy control levels (13). In our study, the initial higher cg02652579 methylation showed a tendency of decreasing towards control levels. Moreover, the general tendency of *SYNGAP1* DNAm reversal after withdrawal therapy is supported by the findings of Witt et al., who identified two other CpG sites within the *SYNGAP1* gene body, whose methylation levels changed with therapy: cg01069468 (first intron) and cg26257411 (third intron), both of which were higher methylated post treatment compared to prior (37).

Taken together, we were not able to validate the findings of Brückmann et al. (2017) in our study. The opposite direction of alteration observed in our recent data could be attributed to differences in the study materials analyzed, as DNAm varies widely across tissues (38). This is further supported by our data from whole blood and saliva that show different methylation levels of the same CpG site within the same individuals. It is plausible that *SYNGAP1* DNA methylation does not exhibit a uniform pattern of dysregulation across tissues in AUD, but instead reflects heterogeneous or context-specific changes. Furthermore, Brückmann et al. studied DNAm in a cohort only consisting of males. Therefore, even if the AUD diagnosis is the same in male and female patients in our cohort, different drinking patterns may induce differential DNAm of *SYNGAP1*. For example, women with AUD may demonstrate a telescoping pattern—initiating drinking later than men but advancing more rapidly to dependence and treatment in clinical samples (39). Furthermore, due to sex-specific biological differences in alcohol metabolism (e.g., lower total body water, reduced dehydrogenase activity), women tend to reach higher blood alcohol levels than men from equivalent intake and are more prone to harm, even at lower drinking levels (40, 41). However, males and females revealed no differences in *SYNGAP1* DNAm in our cohort.

SynGAP, encoded by *SYNGAP1*, plays a central role in excitatory synaptic networks, including the postsynaptic density and NMDAR complexes, where it regulates excitability and plasticity (13–16, 38, 39). Because the analyzed CpG site is located in the promoter region, higher DNA methylation could suppress *SYNGAP1* expression (42, 43), possibly leading to reduced SynGAP protein and downstream signaling changes involving Ras/Rab/Rap, ERK, and AMPAR insertion (15, 17, 18, 20–22, 44). This may hypothetically resemble chronic ethanol effects, which have been linked to altered NMDAR activity, AMPAR expression, and increased hippocampal excitability in rodents (45–52). These interpretations remain highly speculative and require direct experimental validation. While direct evidence linking *SYNGAP1* DNAm to AUD symptoms remains limited, dysregulation of synaptic gene methylation is increasingly recognized in AUD pathophysiology (53, 54). Studies have shown that alcohol exposure alters DNA methylation in genes related to synaptic function and neuronal communication, which may influence AUD-related behaviors (55). Although *SYNGAP1* methylation itself has not been extensively studied in the context of AUD, its role in synaptic plasticity suggests potential involvement in molecular mechanisms underlying addiction and symptom severity. Further targeted studies are warranted to explore *SYNGAP1* methylation changes in AUD and their clinical implications.

Epigenetic marks vary fundamentally between individuals and different somatic tissues (56, 57). The choice of tissue and cell type to analyze in order to provide robust information about epigenetic mechanisms concerning the respective research object is substantial and challenging (38). In an online tool created by Hannon et al., a trend of correlation between *SYNGAP1* DNAm in blood and the prefrontal cortex was displayed (r = 0.219, *p* = 0.061 (58)). The prefrontal cortex is especially intertwined in the neurocircuitry of addiction and it is ascribed a central position in the controlling of craving (59). Simultaneously, its activation decreases and impedes decision making and self-regulation (59). Therefore, a neuronal activation during craving would be correlated with an increase in *SYNGAP1* DNAm, which would enable glutamatergic activity. This is coherent with our finding of a significantly higher *SYNGAP1* DNAm in blood of patients compared to control individuals and substantiates the potential as a possible diagnostic biomarker. However, as we, as well as Brückmann et al. (2017), examined peripheral tissues, we are not able to draw final conclusions on the regulation of *SYNGAP1* in the brain of AUD patients through differential DNAm. A potentially tissue dependent epigenetic regulation of *SYNGAP1* is supported by our findings in saliva, where we did not identify any effects of AUD on *SYNGAP1* DNAm. Although in an earlier study, an impact of hazardous drinking behavior on DNA methylation was observed in saliva (60), *SYNGAP1* sites were not among the differentially methylated CpG sites. We therefore conclude that *SYNGAP1* DNAm in saliva cannot be used as a biomarker for AUD diagnosis or therapy outcome. However, saliva DNA methylation analysis faces unique technical challenges, including contamination with bacterial DNA, DNA fragmentation, and variability in cell types, which can affect data quality and sensitivity. Therefore, technical limitations may contribute to the null findings for *SYNGAP1* methylation in saliva, warranting cautious interpretation and further methodological refinement.

Interestingly, Brückmann et al. restricted their analyses to smokers and observed different *SYNGAP1* methylation patterns. Smoking is known to exert widespread epigenetic effects, including changes in DNA methylation across multiple loci (Zillich 2022), which could interact with or mask alcohol-related methylation signals. Thus, differences between studies may partly reflect the inclusion of non-smokers in our sample, highlighting a potential modulatory role of smoking on *SYNGAP1* DNA methylation.

Taken together, this study has several limitations: Given the small sample, the study was likely underpowered to detect effects of small magnitude. Additionally, sample size (especially of patients) decreased from T1 to T2, leading to reduced sample sizes over time. While the longitudinal mixed models applied can accommodate missing data, the smaller numbers remain a limitation for *post hoc* comparisons of change between time points. Moreover, the AUD patient group and the healthy control group were not properly matched concerning age and sex. Although we have examined these variables for their potential to confound our results in a mixed-effects model, hidden effects cannot be excluded. In addition, smoking was assessed as a binary yes/no variable, which may have obscured differences in intensity, duration, or recency of use. This simplification could reduce statistical power, mask dose–response relationships, and introduce residual confounding. Furthermore, smoking status largely overlapped with AUD status. Therefore, it is not possible to distinguish between AUD and smoking and the effects of these variables on *SYNGAP1* DNAm. In order to tackle this problem within our data, we included not only smoking status, but also the interaction of smoking and AUD status (to analyze potential additive or interactive effects) in our linear mixed-effects models, which did not reveal any notable effect. Moreover, in this study, cell-type composition measures were not available for the blood or saliva samples analyzed. As methylation levels can vary substantially across cell types, this represents a potential confounding factor that may influence interpretation of DNA methylation results. While computational deconvolution methods exist for genome-wide methylation data, they are not applicable for targeted, single-gene methylation assays due to limited coverage. Therefore, the effects of cell-type heterogeneity could not be directly assessed or corrected in our analyses. The *SYNGAP1* DNA methylation differences observed in our study (~1%) are substantially smaller than the 6% reported by Brückmann et al., which may limit their potential functional impact; however, a 1% difference in DNAm is small but not necessarily negligible, as its significance depends on CpG location, tissue/cell type, and the biological context of the gene, and for dosage-sensitive neural genes even minor changes could theoretically influence protein levels and downstream signaling. Moreover, gene expression underlies a complex network of regulatory factors (61) of which DNAm represents only one (62). Unfortunately, literature has been limited to gene expression or DNAm of *SYNGAP1*. Investigations into additional mechanisms related to *SYNGAP1* expression represent a necessary topic of research to provide a more complete picture of its regulation in general and specifically in association with alcohol consumption and AUD.

In conclusion, differential DNAm of *SYNGAP1* could not be reliably validated in comparison to the previous study of Brückmann et al. (13) in whole blood, although differential methylation levels were observed when not including potential confounding factors. When extending the analysis to saliva, we observed no differences in *SYNGAP1* DNAm comparing AUD patients and healthy control individuals. We neither observed an effect of withdrawal therapy on *SYNGAP1* DNAm in whole blood, nor in saliva. As the effects in blood were small and there were no effects in saliva, we conclude that *SYNGAP1* DNAm provides restricted potential as a biomarker for AUD diagnosis – perhaps as part of a panel – but not therapy. An important challenge for future studies is the identification of biomarkers with stronger effects in sample materials that meet the requirement for both informative value and convenient access and analysis.

## Data Availability

The original contributions presented in the study are included in the article/**Supplementary Material**. Further inquiries can be directed to the corresponding author/s.
